# Global burden of osteoarthritis and musculoskeletal diseases

**DOI:** 10.1186/1471-2474-16-S1-S3

**Published:** 2015-12-01

**Authors:** Anthony D Woolf

**Affiliations:** 1 University of Exeter Medical School, UK

## 

Over the past century, global health priorities were largely focused on communicable diseases. With the world's population growth, increased average age and decreased death rates, people are now living longer and becoming increasingly susceptible to the non-communicable diseases, including musculoskeletal (MSK) disorders. The recent Global Burden of Disease (GBD) Study estimated the burden disability in 187 countries and 21 regions of the world for the years 1990, 2010 and 2013 of all MSK disorders - osteoarthritis (OA), rheumatoid arthritis (RA), gout, low back pain (LBP), neck pain (NP) and all other musculoskeletal disorders. Throughout the world, the prevalence and burden from MSK conditions were exceptionally high. All MSK disorders combined caused 21.3% of the total years lived with disability (YLDs) globally - second to mental and behavioural problems (23.2%). When taking into account both death and disability, all MSK disorders combined accounted for 6.7% of the total global disability-adjusted life years (DALYs), which was the fourth greatest burden on the health of the world's population (third in the developed countries). Out of the 291 conditions studied, LBP ranked first (highest) for the disability (YLDs), and sixth for the overall burden (DALYs). For NP, the condition ranked fourth highest for YLDs, and 21st for DALYs. ‘Other MSK disorders’ ranked sixth highest for YLDs and 23^rd^ for DALYs. Osteoarthritis, RA and gout were also significant contributors to the global disability burden. In addition to this burden of disability as estimated by these summary measures of health, there is the impact on the individual's quality of life and economic independence as well as the costs to society due to health and social care and due to work loss. Despite this enormous and growing burden there is a lack of priority and of policies focusing on musculoskeletal health. This needs to change if we are to meet the demands of an ageing population that needs to be able to remain economically independent.

